# Lower Bone Mineral Density at the Hip and Lumbar Spine in People with Psychosis Versus Controls: a Comprehensive Review and Skeletal Site-Specific Meta-analysis

**DOI:** 10.1007/s11914-016-0325-0

**Published:** 2016-10-01

**Authors:** Lucia Gomez, Brendon Stubbs, Ayala Shirazi, Davy Vancampfort, Fiona Gaughran, John Lally

**Affiliations:** 1 Kings College London School of Medical Education, Strand, London, WC2R 2LS UK; 2 Health Service and Population Research Department, Institute of Psychiatry, Psychology and Neuroscience, King’s College London, De Crespigny Park, London, Box SE5 8AF UK; 3 Physiotherapy Department, South London and Maudsley NHS Foundation Trust, Denmark Hill, London, SE5 8AZ UK; 4 Department of Rehabilitation Sciences, KU Leuven-University of Leuven, Leuven, Belgium; 5 University Psychiatric Centre KU Leuven, KU Leuven–University of Leuven, Leuven-Kortenberg, Belgium; 6 Department of Psychosis Studies, Institute of Psychiatry, Psychology & Neuroscience, King’s College London, London, UK; 7 National Psychosis Service, South London and Maudsley NHS Foundation Trust, London, BR3 3BX UK; 8 Department of Psychiatry, Royal College of Surgeons in Ireland, Beaumont Hospital, Dublin, Ireland

**Keywords:** Bone mineral density, Schizophrenia, Psychosis, Osteoporosis, Fracture

## Abstract

It remains unclear if differences in bone mineral density (BMD) exist at different skeletal sites between people with schizophrenia and age- and sex-matched healthy controls (HCs). Major databases were searched from inception until February 2016 for studies measuring BMD using dual-energy X-ray absorptiometry (DXA) at any skeletal site in individuals with schizophrenia. Ten studies investigating 827 people with schizophrenia (55.4 % female, 33.8 ± 9.7 years) and 1379 HCs (58.7 % female, 34.7 ± 9.1 years) were included. People with schizophrenia had significantly reduced BMD at the lumbar spine (standardised mean difference adjusted for publication bias (SMD) = −0.950 (95 % CI = −1.23 to −0.66, fail-safe number = 825) and hip (SMD = −0.534, 95 % CI = −0.876 to −0.192, fail-safe number = 186). A higher proportion of hyperprolactinaemia (*β* = −0.0102, *p* < 0.0001) and smokers (*β* = −0.0099, *p* = 0.02) moderated a larger reduced BMD at the lumbar spine. Further research is required to investigate if low bone mass and fractures can be prevented in people with schizophrenia.

## Introduction

Osteoporosis is a progressive disease characterised by a marked loss as well as a change in the microstructure of bone tissue, resulting in a weakening of the skeletal structure [1]. Its precursor, osteopenia, although not a disease category in itself, also carries an increased risk of fracture [1]. Worldwide, 200 million people are estimated to have osteoporosis [2]. The fragility fractures commonly resulting from this condition are associated with an increase in morbidity and mortality, as well as a decrease in overall quality of life [3]. People with schizophrenia experience poorer general health outcomes than the general population, including an increased risk of osteoporosis [4••]. The mechanism underlying reduced bone mineral density (BMD) in schizophrenia is not yet clear, and links have been made to lifestyle factors as well as the side-effects of antipsychotic medication [5, 6]. Nonetheless, people with schizophrenia are at increased risk of fractures [7] and in particular hip fractures [8•], which are associated with considerable morbidity and mortality.

It has been suggested that antipsychotic-induced hyperprolactinaemia (defined as a serum PRL >24 ng/ml for females and a level >20 ng/ml for males) could play a role in the development of reduced BMD in people with schizophrenia [9]. Other risk factors for osteoporosis such as smoking, excessive alcohol consumption, lack of physical activity, diabetes and vitamin D deficiency are also more prevalent in people suffering from psychotic illnesses [10, 11•] and likely contribute to the development of osteoporosis in this patient group.

A recent meta-analysis demonstrated that over half (51.7 %) of people with schizophrenia have low BMD equating to an almost threefold increased risk versus healthy controls (odds ratio (OR) = 2.86, CI = 1.27–6.42, *p* = 0.01 [4••]). This increased prevalence of osteoporosis and osteopenia in patients with schizophrenia is of particular concern because of their increased risk of falls [12] and fractures, which can have a serious negative impact on the patient’s mental state [4••]. Patients with schizophrenia have a longer recovery time after hip fracture, spending on average 11 days more in hospital compared with those with no mental health conditions [13••] and are at a greater risk of adverse events such as postoperative infection, deterioration of mental state and renal failure [13••]. The time to full rehabilitation is also increased, with ambulatory rates below average after 1 year [14]. A recent meta-analysis [15] demonstrated that people with schizophrenia have reduced BMD, but the authors did not investigate skeletal site-specific differences in BMD and instead pooled all skeletal site results together. Given the particularly serious adverse events following hip fracture, understanding if site-specific differences in BMD exist is important and currently lacking in the literature.

Given the aforementioned gaps within the literature, we conducted a large-scale systematic review and meta-analysis to investigate differences between skeletal sites (e.g. hip, lumbar spine) affected by osteoporosis and osteopenia in people with schizophrenia versus healthy controls. In addition, we sought to identify potential moderators of reduced BMD at skeletal sites in schizophrenia, including gender, age, smoking, prolactin (PRL) levels and body mass index (BMI).

## Method

This systematic review adhered to the MOOSE guidelines [16] and PRISMA statement [17].

### Inclusion and Exclusion Criteria

We included observational studies of any design measuring BMD in both a patient group with a confirmed diagnosis of schizophrenia according to the Diagnostic and Statistical Manual of Mental Disorder [18] or the International Classification of Diseases [19] and a control group of age- and sex-matched people with no diagnosis or history of mental illness. The primary outcome was BMD captured by dual-energy X-ray absorptiometry (DXA) scans. The data were collected as *T*-scores, which compare the measured BMD with the mean for a young adult of that gender, or *Z*-scores, which compare the measured BMD with that of an age- and gender-matched mean value. For studies which reported both *T*- and *Z*-scores, we used the *T*-score as recommended by the World Health Organisation to better predict risk of fracture [20]. The two studies reporting only *Z*-scores [21, 22] were performed in a young adult population for whom *T*- and *Z*-scores are approximately equivalent. As such, we used *Z*-scores in young adult populations when it was the only measure reported. One study [23] reported BMD in grams per square centimeter in both comparative groups. Meta-analysis calculating standardised mean differences (SMD) enables pooling of different unit measures of the same outcome to be standardised and compared with each other, such as Z- and T-scores.

For studies which used the same sample group of patients at a different point in time, we used data from the study with the largest data set or the most recent study. Studies without a control group with no diagnosis of mental illness were excluded from this review. For studies which included a patient group with a mixed diagnosis (e.g. schizophrenia and bipolar disorder), we contacted the authors to request the schizophrenia-specific data, although we did not receive this data from the authors we contacted and subsequently excluded one study [24].

Studies using quantitative ultrasound scanning (QUS) were excluded from the meta-analysis. Although the more portable nature of QUS scanners makes it an appropriate method of evaluating BMD in large populations, QUS lacks sensitivity and specificity when compared with DXA and so cannot be used as a direct alternative to DXA scanning [25]. QUS also provides a different measure of bone structure to DXA scans [26].

### Literature Search and Study Selection

Two independent authors (L.G. and B.S.) systematically searched PsycINFO, Medline, PubMed, Embase, AGRIS and PsychARTICLES from inception until February 2016 using the following search terms: (osteoporosis or osteopenia or osteo* or BMD or DXA or DEXA) and (schizophrenia or schizo* or psychosis or antipsychotics) and with removal of duplicate articles. The reference lists of articles that met the inclusion criteria and those obtained from other relevant systematic reviews on this topic [15, 27–29] were subsequently reviewed for any further suitable studies. The corresponding authors of research groups were contacted where additional information was necessary, but none provided further information. Two authors (L.G. and B.S.) independently searched through titles, abstracts and full-text articles for review. Duplicate articles were removed. Articles deemed viable were cross-checked by both authors and an independent reviewer (J.L.), to ensure that they met the inclusion criteria.

### Data Extraction

Two authors (L.G. and B.S.) extracted data from the relevant studies including region, and details of the participants (% female, mean age, antipsychotic prescribed, PRL levels, mean body mass index (BMI), % smokers), alongside the relevant control group characteristics. We also extracted details of the methods used to scan for BMD including skeletal site and type of scan used. For studies which provided this information, mean levels of vitamin D and PRL were recorded. PRL levels for one study [21] were obtained from a parallel study reporting PRL levels in the same patient cohort at the same point in time [30]. The data were collected in a predetermined database.

### Data Analysis

We compared BMD differences between people with schizophrenia and healthy controls at each different skeletal site by calculating the SMD together with 95 % confidence intervals (95 % CI). Effect sizes were classified as small (*d* = 0.2–0.49), medium (*d* = 0.5–0.79), and large (*d* ≥ 0.8) [31].

Heterogeneity was assessed using the *I* ^2^ statistic. Scores of over 75 % were classed as high heterogeneity, suggesting that the differences in aggregate BMD are larger than can be expected based on random error alone. Publication bias was assessed through a visual funnel plot, and the Begg [32] and parametric Egger [33] tests. The analyses were adjusted for publication bias and outliers using the Duval and Tweedie trim and fill analysis [34]. We also investigated, where possible, potential moderators using meta-regression analysis. The moderators of interest were mean age, per cent females, per cent taking antipsychotic medication, mean PRL levels, per cent hyperprolactinaemia, smoking rates, mean BMI, control mean age and control per cent female. All analyses was conducted with Comprehensive Meta-analysis V3 (CMA v3, Englewood, NJ, USA).

### Results

**Fig. 1:**
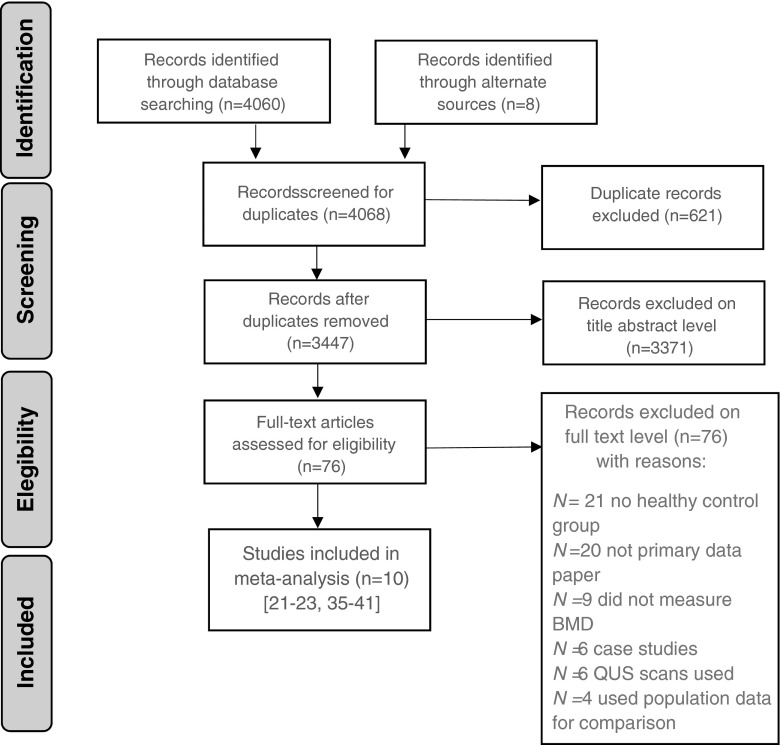
PRISMA search results

The initial search of the available literature yielded 4060 articles. After removal of any duplicate articles, 3447 titles and abstracts were screened, of which 76 were assessed at full-text level for suitability for inclusion. Of these, ten met the necessary criteria for inclusion in the meta-analysis [21–23, 35–41]. Two studies did not provide the full details of BMD measurements [42, 43]. Four studies reported BMD measurements for a patient group with schizophrenia but used population data rather than an age- and sex-matched control group [28, 44–46]. We contacted the authors of these studies to request further information, but no study group provided this information and they were excluded due to insufficient data. Full details of the search results and reasons for exclusion are presented in Fig. 1.

The patient group included 827 people with schizophrenia in total, and an age- and sex-matched control population of 1379 people without any current or history of mental illness. The mean age of participants with schizophrenia was 33.8 years (SD = 9.7 years) and 55.4 % were female. The mean age of the control group was 34.7 years (SD = 9.1 years) and 58.7 % were female. Seven studies were conducted in Europe [21–23, 36, 37, 40, 41], and three in Asia [35, 38, 39]. Sample sizes ranged from 14 [37] to 229 [38]. All but one of the included studies were cross-sectional, reporting a single measurement of BMD. There was one prospective study [35], measuring BMD before and after treatment with either atypical or typical antipsychotic medication. For this study, we only included the baseline data (pre-treatment atypical and typical antipsychotic) arms before antipsychotic treatment began. Ten studies measured BMD at the lumbar spine [21–23, 35–41] of which five also measured BMD at the hip [22, 23, 37–39]. Information regarding potential risk factors for osteoporosis was varied, with data on PRL levels available in four studies [21, 22, 35, 39] and vitamin D levels reported by only one study [22]. Although all studies provided information on the class of antipsychotic used in each patient subgroup, only five gave details of the antipsychotics used [21, 22, 35, 39, 41]. Details on the included studies and the participants are presented in Table 1.

**Table 1:** Summary of included studies

c	Study name	Design and setting	Subjects		Total *N* in group	*N* in subgroup	Primary outcome	Value	Mean age	Sex (% female)	Details of antipsychotics	Prolactin (ng/ml)	% hyperprolactinaemia	Oestrogen (pg/ml)	BMI	% smokers
[29]	Wyszogrodzka et al. (2015)	Poland (cross-sectional; inpatient)	Patient	M risperidone	60	10	*Z*-score	−0.6 ± 0.74	31.1 ± 8.7	60	26 risperidone	44.7	90	n/a	n/a	n/a
				F risperidone		16		−0.16 ± 1.13			34 olanzapine	81.1	93.8			
				M olanzapine		14		−0.95 ± 1.50				21.9	85.7			
				F olanzapine		20		−0.47 ± 1.16				29.8	70			
			HC	M	38	17		−0.15 ± 1.12	31.7 ± 8.0	55.2		10.8	0	n/a	n/a	n/a
				F		21		0.47 ± 0.8				16.1	4.8			
[32]	Wang et al. (2014)	China (prospective; community)	Patient	PR, pretreatent	163	81	*T*-score	1.26 ± 0.19	34.5 ± 10.7	47.5	26 clozapine	31.73	22.18	45.23	25.7	n/a
				PR, post-treatment				1.23 ± 0.10			28 quietapine	53.05	30.25	22.87		
				PS, pretreatment		82		1.14 ± 0.15			28 aripiprazole	29.79	16.03	47.02	26.8	
				PS, post-treatment				1.16 ± 0.18			26 chlorpromazine	32.81	17.42	45.99		
											27 perphenazine					
											28 sulpiride					
			HC	M	90	47		1.28 ± 0.15	34.2 ± 10.6	48		n/a	n/a	n/a	25.3	n/a
				F		43		1.28 ± 0.13								
[33]	Van Der Leeuw et al. (2013)	Netherlands (cross-sectional; mixed)	Patient	M femoral	54	46	*T*-score	−0.58 ± 1.30	27.4 ± 6.4	25.8	3 haloperidol	n/a	n/a	n/a	24.2	n/a
				M L spine				−0.235 ± 1.07			1 zuclopenthixol					
				F femoral		16		−0.369 ± 0.95			17 risperidone					
				F L spine				−0.125 ± 1.02			7 amisulpride					
											*No further details*					
			HC	M femoral	62	46		−0.09 ± 0.75	31.2 ± 11.4	72.9		n/a	n/a	n/a	24.31	n/a
				M L spine				−0.57 ± 1.32								
				F femoral		16		0.15 ± 0.99								
				F L spine				0.10 ± 1.19								
[34]	Maric et al. (2005)	Serbia (cross-sectional; mixed)	Patient		19	19	g/cm^2^	1.13 ± 0.10	23.7 ± 3.1	100	Short exposure (mean 10 weeks)	n/a	n/a	n/a	n/a	58
											*No further details*					
			HC		39	39		1.25 ± 0.12	24.5 ± 3.8	100						n/a
[35]	Kocer et al. (2011)	Turkey (cross-sectional; outpatient)	Patient	L spine	14	14	*T*-score	−0.6 ± 1.40	33.1 ± 9.0	35.7	8 PRs	n/a	n/a	n/a	n/a	60
				Femoral				−0.66 ± 0.99			6 PS					
											*No further detail*					
			HC	L spine	45	45		0.2 ± 1.15	34.4 ± 7.0	51.6		n/a	n/a	n/a	n/a	n/a
				Femoral				0.16 ± 1.08								
[36]	Jung et al. (2011)	South Korea (cross-sectional; inpatient)	Patient	M L spine	229	136	*T*-score	−0.15 ± 1.07	58.7 ± 6.8	40.6	207 PR	n/a	n/a	n/a	23.1	55.1
				M femoral				−0.02 ± 1.06			22 PS					
				F L spine		93		−0.13 ± 1.08			*No further details*				22.3	14
				F femoral				−0.81 ± 0.91								
			HC	M L spine	125	65		0.16 ± 1.13	58.6 ± 6.4	48		n/a	n/a	n/a	21.9	13.8
				M femoral				0.4 ± 0.87								
				F L spine		60		0.74 ± 1.01							22.5	3.3
				F femoral				0.21 ± 1.15								
[37]	Jung et al. (2006)	South Korea (cross-sectional; inpatient)	Patient	M	51	30	*T*-score	−0.14 ± 1.06	38.9 ± 5.3	41.1	All halperidol monotherapy	22.2	40	37.2	23.9	0
				F		21		−0.73 ± 1.16				69.5	90.5	52.4	23.5	50
			HC	M	57	34		0.39 ± 1.11	38.6 ± 4.9	40.4		n/a	n/a	n/a	24.1	0
				F		23		−0.13 ± 1.13							22.8	38.2
[38]	Doknic et al. (2011)	Serbia (cross-sectional)	Patient	L spine	26	26	*Z*-score	−0.561 ± 1.31	31.3 ± 1.3	53.8	All long-acting injectable risperidone	81	84.6	148.9^a^	28.2	46
				Femoral				−0.17 ± 1.06								
			HC	L spine	61	61		−0.23 ± 0.99	32.2 ± 1.4	68.6		16.5	n/a	347.5	28.1	20
				Femoral				−0.07 ± 1.03								
[39]	Bilici et al. (2011)	Turkey (cross-sectional; inpatient)	Patient	PR	136	94	*T*-score	−0.88 ± 0.9	39.4 ± 7.6	100	No further details	n/a	n/a	n/a	29.6	n/a
				PS		42		−0.99 ± 0.9							28.2	
			HC		276	276		−1.14 ± 0.8	39.4 ± 8.4	100					28.7	n/a
[40]	Bilici et al. (2009)	Turkey (cross-sectional; outpatient)	Patient	PR	75	40	*T*-score	0.94 ± 0.21	29.7 ± 6.5	49.3	20 halperidol	n/a	n/a	n/a	27.2	n/a
				PS		35		1.06 ± 0.52			10 fluflenazin				33.4	
											10 zuclopentixol					
											10 olanzapine					
			HC		95	95		1.08 ± 0.49	31.1±7.1	50	20 risperidone	n/a	n/a	n/a	26.7	n/a
											5 clozapine					

Data presentation: mean ± SD

*N* number, *BMI* body mass index, *HC* healthy control, *M* male, *F* female, *PR* PRL raising, *PS* PRL sparing, *n/a* not available

^a^Females only

## Meta-analysis

### Lumbar Spine Bone Mineral Density

**Fig. 2:**
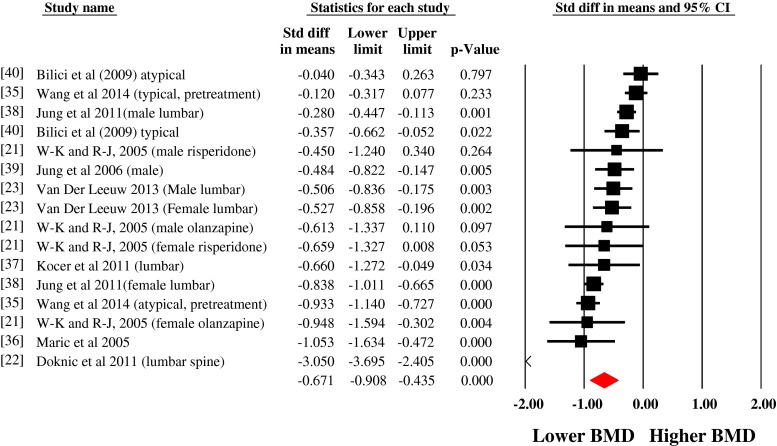
Meta-analysis of the differences in bone mineral density at the lumbar spine in schizophrenia versus controls

The pooled BMD estimates from ten studies showed that people with schizophrenia have a reduced BMD at the lumbar spine (SMD = 0.671, 95 % CI, −0.908 to −0.435), consistent with a medium effect size (see Fig. 2). There was evidence of heterogeneity (*I* ^2^ = 88 %). Neither the Begg (Tau = −0.22, *p* = 0.22) nor Egger tests (−1.79, *p* = 0.23) showed evidence of significant publication bias. The Duval and Tweedie trim and fill methods were used to adjust for publication bias and outliers. This established that people with schizophrenia have an even greater reduction in lumbar spine BMD with an effect size of −0.950 (95 % CI, −1.23 to −0.66). The fail-safe number of studies (i.e. the number of negative studies to take *p* > 0.05) was high at 825.

### Meta-regression of Moderators of Lumbar Spine BMD Differences

Full details of the meta-regression analyses are presented in Table 2. Briefly, a higher number of people with hyperprolactinaemia (*β* = −0.0102, 95 % CI, −0.0135 to −0.0068, *p* < 0.0001) moderated a greater reduction BMD at the lumbar spine in those with schizophrenia compared with controls. There was also evidence that an increased prevalence of smoking in schizophrenia (*β* = −0.0099, 95 % CI, −0.0185 to −0.0012, *p* = 0.02) moderated a greater reduction in BMD at the lumbar spine in schizophrenia compared with controls.

**Table 2:** Meta-regression of bone mineral density differences between schizophrenia and control participants

Moderator	Number studies estimates	*β*	95 % CI		*P* value	*R* ^2^
Lumbar Spine BMD results						
Mean age	14	−0.0019	−0.0368	0.0331	0.9173	0
% females	14	0.0029	−0.0059	0.0117	0.5224	0.03
% taking antipsychotic medication	9	−0.0062	−0.0182	0.0058	0.3085	0.09
Serum prolactin	7	−0.0077	−0.0175	0.0022	0.1268	0.31
% people schizophrenia hyperprolactinaemia	7	−0.0102	−0.0135	−0.0068	*<0.0001*	1.0
Body mass index	11	−0.0148	−0.103	0.0734	0.7423	0.01
% people schizophrenia who smoke	6	−0.0099	−0.0185	−0.0012	*0.0253*	0.61
Control mean age	14	−0.0043	−0.0404	0.0318	0.815	0.01
Control % female	14	−0.0006	−0.0124	0.0112	0.9259	0
Hip BMD results						
Mean age	7	−0.0253	−0.0426	−0.008	0.0041	0.71
% female	7	0.0039	−0.0027	0.0105	0.251	0.21
% taking antipsychotic medication	7	−0.04	−0.1329	0.0529	0.3983	0.11
Body mass index	6	−0.0107	−0.1429	0.1215	0.8739	0

Italic indicates statistically significant result

### Femoral Hip Bone Mineral Density

**Fig. 3:**
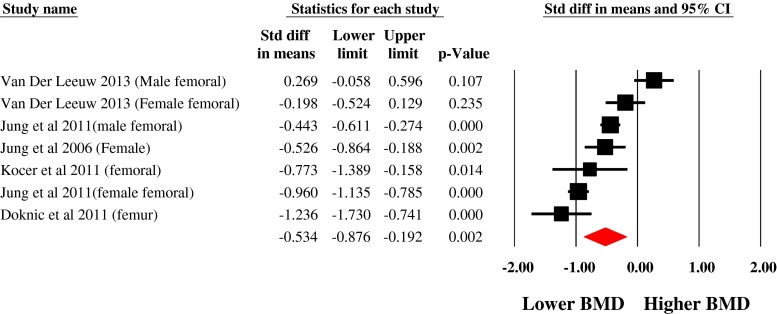
Meta-analysis of the differences in femoral bone mineral density in schizophrenia versus controls

Data from seven estimates across five unique studies demonstrated that people with schizophrenia have a significantly reduced femoral BMD compared with controls (SMD −0.534, 95 % CI, −0.876 to −0.192). There was evidence of heterogeneity (*I* ^2^ = 86 %). The Egger test (1.0, *p* = 0.75) and Begg test (tau = −0.09, *p* = 0.76) demonstrated no significant publication bias. The effect size remained unchanged after calculating the Duval and Tweedie trim and fill analysis. The fail-safe number of studies required to null the difference was high at 168 (Fig. 3).

### Meta-regression of Moderators of hip BMD Differences

There was evidence that an increase in age (*β* = −0.0253, 95 % CI, −0.0426 to −0.008, *p* = 0.0041) moderated a significantly reduced BMD at the hip in people with schizophrenia compared with healthy controls. Details of the meta-regression analysis are presented in Table 2.

## Narrative Results

### Studies Investigating Hormone Levels and BMD in Schizophrenia

Jung et al. [39] conducted a study amongst 51 medicated in patients with schizophrenia in which all patients were treated with haloperidol monotherapy. Hyperprolactinaemia was evident in 90.5 % of female patients compared with 40 % male patients. The BMD measurements of female patients (*T*-score, −0.73 ± 1.16) were significantly lower than those of males (*T*-score, −0.14 ± 1.06, *p* = 0.008), and a higher proportion of female than male patients showed a reduced BMD (85.7 % female vs. 50.0 % male, *p* = 0.008). In the control group, the gender difference in BMD loss was not significant (39.1 % women vs. 47.1 % men with BMD loss). Significantly higher PRL levels were found in females with BMD loss compared with those with normal bone density. Eighty per cent of female patients had abnormal menstruation (oligomenorrhea or amenorrhea), which was found that all of these also had hyperprolactinaemia. Sustained amenorrhea has previously been identified as a risk factor for osteoporosis [47]. This study from Jung et al. found no significant correlation between BMD and PRL, follicle stimulating hormone (FSH), luteinising hormone (LH), thyroxine (T_4_) or thyroid-stimulating hormone (TSH).

Wang et al. [35] reported BMD before and after 12 months of treatment with first- or second-generation antipsychotics (FGAs or SGAs) in 163 patients with first-episode psychosis (FEP). There was no significant difference in BMD between those with FEP and the healthy control group before treatment with antipsychotics. This study found that BMD decreased from 1.26 ± 0.19 to 1.23 g/cm^2^ across the 12-month treatment period for patients taking FGAs, whereas no significant changes were seen in patients treated with SGAs or in the control group. PRL levels increased over this 12-month period for patients using FGAs (31.73 ± 22.16 ng/ml before, and 53.05 ± 30.25 ng/ml after treatment). FGAs used were chlorpromazine, perphenazine and sulpiride, all of which are PRL raising. There was a small increase in PRL for patients using SGAs (29.79 ± 16.03 ng/ml before and 32.81 ± 17.42 ng/ml after treatment). SGAs used were clozapine, quetiapine and aripiprazole, none of which have a high propensity to increase PRL. The decrease in BMD was correlated with longer treatment periods in the high-PRL group, rather than with PRL levels, suggesting that the duration of hyperprolactinaemia may have a greater impact than PRL levels themselves. Levels of oestrogen decreased after treatment with FGAs (45.23 ± 10.77 U/l before, 22.87 ± 12.14 after; *p* < 0.05), but little change was seen after treatment with SGAs (47.02 ± 14.31 U/l before, 45.99 ± 17.9 U/l after; *p* < 0.05). It is interesting to note that, for the high-PRL group, levels of FSH and LH were decreased and levels of oestrogen also showed a significant decrease, supporting the hypothesis that high-PRL levels inhibit the hypothalamic-pituitary-gonadal axis and thus that antipsychotic medication may play a role in the development of osteoporosis in patients with schizophrenia.

### Studies Investigating Familial Risk of Psychotic Disorders and Low BMD

Van der Leeuw et al. [23] investigated BMD in a patient group, control group and sibling group. In comparison between patients and controls, reduced BMD was found across all measurements in the lumbar spine and hip, although this did not reach statistical significance. In the patient-sibling comparison, there were significant differences found in total BMD and *Z*-score at the femur (*B* = −0.061, 95 % CI, −0.117 to −0.005, *p* = 0.033; and *B* = −0.414, 95 % CI, −0.827 to −0.001, *p* = 0.050, respectively). BMD at the femur was significantly reduced in female patients compared with controls and siblings, with no significant differences found between siblings and controls (*p*_Simes_, *p* < 0.017). In this study group, 6 of the 16 female patients were on a PRL-raising antipsychotic, a factor which was negatively associated with BMD at the lumbar spine when compared with unmedicated female patients (total BMD = −0.113, 95 % CI, −0.220 to −0.006, *p* = 0.041; *Z*-score = −1.109, 95 % CI, −2.243 to 0.025, *p* = 0.054; *T*-score = −1.199, 95 % CI, −2.236 to −0.162, *p* = 0.027). No such correlation was found in male patients.

### Studies Investigating Peak Bone Mass

Maric et al. [36] investigated the BMD of 19 young female patients (24.5 ± 3.8 years) presenting acutely with a first episode of non-affective psychosis. The duration of antipsychotic treatment prior to BMD assessment was on average 10 weeks (range 2–30 weeks). This study found that, on first presentation, patients with schizophrenia had accrued a lower overall bone mass than healthy females of the same age. Eighty-four per cent of patients had values below the median BMD control value (OR = 5.3, 95 % CI, 1.2, 24.2, *p* = 0.030), and results remained similar after the exclusion of the single highest control value. There was a reduced standardised mean difference between patients and controls (patients, 1.13, SD = 0.10 and controls, 1.25, SD = 0.12, *t* = 3.3, *p* = 0.0021). No association was found between duration of antipsychotic treatment and BMD in this study, but the treatment length was short for all patients.

### Studies Investigating BMD Patients Over 50

Jung et al. [38] found an increased prevalence of osteoporosis in 229 patients with schizophrenia aged 50 or older compared with healthy age-matched controls (34.9 vs 18.4 %, *p* = 0.0043), with lower *T*-scores found across all skeletal sites. There was a significant gender difference, with 48.4 % of females with schizophrenia showing evidence of osteopenia or osteoporosis compared with 25.7 % males (*p* = 0.0014). There were no sex differences in prevalence of low BMD in the healthy control group.

## Discussion

To the authors’ knowledge, this is the first meta-analysis to investigate reduced bone mineral density in people with schizophrenia at different skeletal sites. Data from our meta-analysis found a significantly reduced BMD at both the lumbar spine and hip in patients with schizophrenia, with the lumbar spine showing the most marked decrease in BMD, with a large adjusted effect size. The lumbar spine shows reduced BMD in a younger patient cohort before the onset of osteopenia at other skeletal sites. This difference in BMD between skeletal sites may indicate that the lumbar spine is a better site at which to carry out screening for osteoporosis in patients with schizophrenia. Our meta-regression analysis suggests that hyperprolactinaemia and smoking are associated with reduced lumbar spine BMD in people with schizophrenia. In addition, increasing age was associated with greater hip BMD loss.

Fractures of the vertebrae are associated with pain and disability in addition to reduced quality of life and functioning in the general population [48]. Our results demonstrate that people with schizophrenia may be particularly at risk of vertebral fractures, the principle symptom of which is pain [49•]. People with schizophrenia have greatly reduced pain sensitivity [50] meaning that in clinical practice, patients with a vertebral fracture may be less likely to report symptoms. Given this fact and the heightened risk of vertebral fractures, clinicians should be diligent in their assessment of back pain in people with schizophrenia and an observed thoracic kyphosis may be a possible indicator of fracture in this population [51].

People with schizophrenia experience a higher mortality, longer hospital stays and poorer recovery of mobility following a hip fracture, as well as a negative impact on mental state caused by hospitalisation [4••]. The presence of reduced BMD at the hip in patients with schizophrenia is of particular concern because of the more severe clinical implications of fracture for this patient group compared with patients without a history of mental illness. Considering the greatly increased risk of fracture for these patients [7], it may be advisable to introduce a screening programme to monitor bone health and thus improve general health outcomes in this patient population.

Meta-regression analysis of moderators of low bone mass highlighted smoking as a risk factor for reduced BMD at the lumbar spine (Table 2). People with schizophrenia have high rates of smoking with higher cigarette consumption amongst those who smoke [52, 53], factors which can contribute to the development of osteoporosis [54, 55•]. The effects of smoking on BMD found in this study further indicate the need to develop effective interventions for smoking cessation amongst those with schizophrenia.

Antipsychotic medication was identified as a risk factor for osteoporosis in the narrative review, with PRL-raising antipsychotics having a larger impact on bone health than PRL-sparing medication. Of the four studies included in this review, investigating PRL alongside BMD, all found elevated levels of PRL in at least one patient group [21, 22, 35, 39]. Two of the four studies [21, 35] investigated patients on monotherapy with PRL-raising or PRL-sparing antipsychotics and found higher PRL levels in the PRL-raising group. However, it is important to note that these studies were not carried out in treatment-naïve patients and so previous treatment with a range of antipsychotics may have already had an impact on bone health.

Schizophrenia is usually diagnosed between the ages of 16 and 30, at which point peak bone mass has yet to be achieved [56]. When administered to such a young patient group, PRL-raising medications may prevent an optimum peak bone mass from being achieved, thus reducing lifelong BMD and predisposing patients to osteoporosis. Our study findings should be viewed in relation to the recognition that sustained hyperprolactinaemia can have longer term adverse bone effects, including increasing the risk for osteoporotic fractures [7]. This is in addition to other established risks to bone health such as low vitamin D, commonly seen in psychosis [10, 11•]. Our findings that hyperprolactinaemia is associated with reduced BMD in schizophrenia provides additional support and impetus for the inclusion of regular PRL monitoring in guidelines for physical health monitoring in those with schizophrenia.

The mean age of the schizophrenia cohort in our study was 34 years, with only one study [38] measuring BMD in a patient group older than 50. Osteoporosis is a disease of the older population, most commonly seen in postmenopausal women and men over 65 [3]. The presence of reduced BMD in such a young population as identified in our study is concerning, particularly because of the scale of the BMD loss, which was above 50 %, even with the exclusion of the Jung et al. study [38]. Current UK guidelines suggest assessment of fracture risk in men over 50 and postmenopausal women, and treatment only of high-risk groups above that age [57]. Our study findings indicate that those with schizophrenia are at an increased risk of reduced BMD and suggest that a diagnosis of schizophrenia should be considered alongside other well-validated risk factors for reduced BMD, to improve fracture prognostication in this population, and prompting a lower threshold for the initiation of treatment in this population with increased fracture risk.

For patients younger than this age threshold, it is unlikely that medical intervention to prevent fractures would be considered. It may be necessary to adapt guidelines to manage the risk of osteoporosis in people with schizophrenia, perhaps by introducing a modified *T*-score as a threshold for medical intervention regardless of age. Careful assessment for modifiable risk factors for the development of osteoporosis in schizophrenia such as smoking, hyperprolactinaemia, and vitamin D deficiency, and appropriate treatment interventions should be more fully incorporated into the care of schizophrenia patients. Advice on weight-bearing exercise, calcium and vitamin D intake and smoking cessation should be provided to patients. For individuals with hyperprolactinaemia, consideration of dose reduction or a switch to an antipsychotic with a lower risk of causing hyperprolactinaemia should be made, if clinically safe to do so. If the risk of psychotic relapse is high, then consideration for an addition of aripiprazole to the antipsychotic treatment could be made, as its use has been associated with attenuation of PRL levels [58].

### Limitations

It is necessary to acknowledge the limitations of both the primary data and our meta-analysis. Firstly, due to lack of information, we could not investigate the effects of specific antipsychotic medications on BMD. Few studies separated patients by type of antipsychotic and only two consistently reported the dose used. Although several studies separated subgroups into PRL-raising/PRL-sparing or FGA/SGA subgroups and some provided details of the antipsychotics used, these classifications can overlap in individual antipsychotic effect on PRL, and the potential effects on BMD could be unclear. Further, the lack of information on lifetime antipsychotic use is a major limitation in our ability to examine the enduring effects of antipsychotic use on BMD. Second, the mean age of this patient group was 34 years with little inclusion of older patients, which is not representative of the general patient population. Thirdly, all but one of the studies were cross-sectional in design, with only one [33] investigating BMD in treatment-naïve people with schizophrenia. More studies of this kind may help identify risk factors without the confounding influence of antipsychotic medication. Finally, there was considerable heterogeneity and/or absence in reporting data for risk factors such as smoking, vitamin D, diet, ethnicity, medical comorbidities (e.g. diabetes) and exercise levels, such that although several studies did report this information, we were unable to pool some or all of the data for moderator analysis.

## Conclusion

Our systematic review and meta-analysis have shown a significantly reduced BMD at the lumbar spine and hip in people with schizophrenia. This is of particular concern considering the young average age of this patient cohort, for whom there would be few screening processes and preventative measures in place to reduce the risk of fracture. In recognition of the increased morbidity and mortality associated with fracture for people with schizophrenia, there is a need for further research to ascertain the aetiology of this reduced BMD such that risk factors can be modified and interventions put in place to reduce the risk of fractures in this vulnerable patient group.
